# How work stress influence turnover intention among Chinese local undergraduate university teachers: the mediating effect of job burnout and the moderating effect of self-efficacy

**DOI:** 10.3389/fpubh.2024.1308486

**Published:** 2024-03-19

**Authors:** Shuimei Pei, Shichao Wang, Ruixuan Jiang, Jianpeng Guo, Jianchao Ni

**Affiliations:** ^1^College of Humanities, Xiamen Huaxia University, Xiamen, Fujian, China; ^2^Institute of Education, Xiamen University, Xiamen, Fujian, China

**Keywords:** turnover intention, work stress, job burnout, self-efficacy, moderated mediation

## Abstract

**Background:**

Turnover intention is one of the common disturbances that affect the career development and management of university teachers. With an aim to solve this thorny issue, the study examined the associations between turnover intention and work stress among local undergraduate university teachers in China.

**Methods:**

A questionnaire survey was carried out on 7,565 local university teachers. Initially, confirmatory factor analysis was employed to validate the reliability and validity of the scale. Subsequently, descriptive statistics and correlation analyses were conducted. Following this, a latent moderated structural equation (LMS) was used to explore the relationship among work stress, job burnout, self-efficacy, and turnover intention. Moreover, the bias-corrected Bootstrap method was applied to further examine the mediating effects, moderating effects, and moderated mediating effects in the model.

**Results:**

The hypothesized moderated mediation model was verified significant. Work stress directly and positively predicted job burnout and turnover intention, with job burnout serving a partial mediating role between work stress and turnover intention. Additionally, self-efficacy negatively moderated the direct impact of work stress on job burnout, as well as the mediating effect of job burnout. As the self-efficacy of university teachers increased, the direct effect of work stress on job burnout and the mediating effect of job burnout decreased.

**Conclusion:**

This study expanded the research on the antecedent variables of university teachers’ turnover intention and revealed the individual contingency mechanism by which work stress affected turnover intention: the negative moderating effect of self-efficacy. Work stress induced university teachers’ turnover intention, and this phenomenon was more obvious for faculties with low self-efficacy. Administrators of local undergraduate universities need to rationalize the allocation of teachers’ job responsibilities and pay attention to the negative consequences of work stress in order to reduce turnover intentions. Particular attention should be paid to enhance teachers’ self-efficacy. The findings of this study can provide in-depth recommendations for university faculty management and policy making, which can help shape a working atmosphere more conducive to teaching and research, thus enhancing the overall quality and competitiveness of faculty members within universities.

## 1 Introduction

The current academic discourse in China places significant emphasis on the work stress experienced by higher education faculty, particularly in the urgent and intricate task of mitigating teacher turnover intentions. Teacher turnover intentions serve as a crucial predictor of faculty departure behavior, and an in-depth analysis of the mechanisms underlying these intentions forms the bedrock for the formulation of targeted policies.

Local undergraduate universities in China constitute a crucial component of the country’s higher education system, accounting for over 90% of the total number of higher education institutions nationwide. According to statistics from the Chinese Ministry of Education, there are 1,239 ordinary undergraduate institutions across the country, with an enrollment of over 4.67 million undergraduate students and a faculty consisting of 1.3158 million full-time teachers. These figures emphatically underscore the significant role played by local undergraduate institutions as the backbone of China’s higher education, contributing indispensably to the high-quality development of the regional economic and social sectors.

The key to the advancement of educational endeavors lies in the establishment of a high-caliber and stable teaching faculty, serving as a fundamental element for local undergraduate universities to achieve high-quality development. However, the unprecedented challenges faced by faculty are apparent with the transformation of higher education functions and reforms in personnel systems. Shifts in teaching, research, and societal service responsibilities place multiple burdens on faculty, thereby augmenting overall work stress. Additionally, the profound reforms in personnel systems introduce mechanisms such as performance assessments, even title demotions, contributing to a more stringent evaluation framework for faculty. The cumulative impact of such pressures may detrimentally affect faculty job satisfaction and foster turnover intention, consequently influencing the ability of local undergraduate universities to attract and retain excellent talents. Therefore, an in-depth understanding of the impact of work stress on turnover intention of teachers in local undergraduate universities is crucial to the formulation of effective management strategies.

## 2 Literature review and research questions

The past research has extensively investigated the relationship between work stress and turnover intention among employees in various industries. For instance, Mochamad Soelton et al. (1) analyzed the factors affecting the turnover intention of waiters in chain restaurants. Tahira Nazir et al. (2) studied the moderating and mediating role of organizational cynicism and self-efficacy in the relationship between work stress and turnover intention of medical workers in Pakistan. Lijin Shao et al. (3)used the JDCS model to study the relationship between psychological contract, work stress, and turnover intention of employees in information service companies. Yu-Hui Hsieh et al. (4) explored the mediating role of self-efficacy between job bullying and turnover intention among nurses in Taiwan.

In the education sector, previous studies have shown that there are various explanatory variables for teacher turnover intention (5), such as low school management level, insufficient professional support, and teachers’ lack of professional autonomy (6). Multi-track teaching in schools, overly tight teaching schedules, and daily teaching starting too early or ending too late are all indications of low levels of instructional management. Teachers working in schools with a low level of instructional management have a strong desire to leave and a high turnover rate. In addition, the lower the salary income, the stronger the teachers’ turnover intention (7). These studies have played a positive role by providing insights into turnover intention. However, there is a relative scarcity of articles focusing on teachers in local universities in China in the existing literature.

Moreover, although previous studies have revealed many predictors of turnover intention, research on the role of work stress in inducing turnover intention is still limited. Excessive stress can have a series of negative impacts on teachers’ professional lives, including reduced job satisfaction, organizational commitment, job performance (8), and increased burnout, absenteeism, turnover intention (9). The environmental factors that elicit an individual’s stress can be referred to as stressors. External events become stressors only when people perceive them as threatening or challenging. The main sources of stress for university teachers include teaching and research conflicts, excessive workload, promotion difficulties, and lack of opportunities to participate in management. These stressors cause faculty to perceive stress, which in turn produces a range of emotional, attitudinal, and behavioral responses. Given the prevalence of work stress among university teachers, it is necessary to pay attention to whether this prevalence is an important trigger of turnover intention. Therefore, this study aims to investigate in depth the relationship between work stress and turnover intention among teachers in local universities in China, and to fill the research gap in the current literature.

The Conservation of Resources (COR) theory is employed to elucidate how individuals balance resource inputs and outputs when coping with stress and making decisions. In the realm of higher education, this theory can be applied to analyze the relationship between teachers’ work stress and their turnover intention. Faculty members are required to invest substantial time and effort in fulfilling teaching, research, and service responsibilities. Meanwhile, factors such as educational policies and changes in school management may cause faculty members to feel pressure in various aspects, such as teaching pressure, research pressure, and administrative affairs pressure. If teachers perceive their workload as excessively stressful, and the corresponding outputs, such as salary and job satisfaction, are insufficient to compensate for these inputs, their turnover intention may increase. Resources, in this context, encompass not only economic aspects but also include time, emotions, and social support. The COR theory aids in analyzing the balance between different types of resource inputs and outputs. The theory also emphasizes the adaptation of individual teachers to environmental changes. The COR theory allows us to understand how teachers adjust their resource inputs to change and to analyze the impact of this adaptation on their willingness to leave. Furthermore, the COR theory considers an individual’s long-term development, emphasizing the balance in the accumulation and depletion of resources. In the field of higher education, faculty career development is closely related to work stress and turnover intention. Thus, the COR theory provides a robust theoretical framework for comprehending the intricate relationship between work stress and turnover intention among teachers in higher education.

Then how and when does the work stress of teachers in local undergraduate universities in China affect their turnover intention? Early theories regarded job burnout as a specific form of work stress, referring to a series of psychological and physiological syndromes manifested as stress responses to long-term emotional and interpersonal tension sources (10). Xu suggested that teacher burnout is an extreme reaction when teachers fail to cope with work stress effectively (11). More scholars argue that there are also distinctions between work stress and burnout. Li posited that burnout is a specific and multidimensional work stress, often accompanied by the development of negative attitudes (12). Bian and Long pointed out that work stress often carries a general connotation, while burnout is more closely associated with specific contexts (13). According to the Conservation of Resources Theory, employees are prone to resource depletion when facing stressful situations, which may stimulate individuals to engage in resource defensive behavioral responses, such as doing work procrastination behaviors that reduce the resource invested in order to reduce resource loss and preserve existing resources (14). Work stress poses a threat to the professional lives of university teachers. They are compelled to expend energy to regulate the perceived threat brought about by work stress. And this self-regulation process will lead to a depletion of self-regulation resources (15). Teachers experiencing job burnout may manifest a range of issues related to ineffective self-regulation, such as avoiding work, ultimately leading to the intention to resign in order to preserve existing resources. Thus, there is a close connection between work stress and the turnover intention. Logically, this should be the case; however, this relationship has not yet been empirically verified among teachers in local undergraduate universities in China. Therefore, this study will investigate how work stress among university teachers affects the their turnover intention through job burnout, providing new empirical support, as well as new perspectives on expanding the research path of work stress.

Additionally, considering the psychological cognitive differences among individual teachers, when facing the same work stress, individuals tend to assess it based on their own cognition. Consequently, there may be significant variations in the assessment results and corresponding strategies among teachers with substantial differences. Self-efficacy refers to an individual’s estimation of their ability to undertake challenging tasks and their expectations of exceeding task completion. In dealing with work stress, the self-efficacy of individual teachers is a crucial factor influencing their responses. Recognized as a personal resource, teachers’ self-efficacy is considered one of the most critical resources (16). Per the principle of resource investment in the COR theory, individuals with ample initial resources possess stronger capabilities to acquire resources and are more likely to engage in resource investments, thereby facing a lower risk of resource loss. Therefore, this study posits that self-efficacy, as a positive psychological resource, facilitates proactive acquisition or construction of new resources, particularly self-regulation resources. This, in turn, helps offset potential over-consumption of resources in dealing with work stress, alleviating the degree of job burnout and consequently reducing the likelihood of intentions to resign. Hence, this study incorporates self-efficacy as a moderating variable to delve into the relationship between work stress and turnover intention. So another theoretical contribution of this study lies in revealing the boundary conditions of the transmission mechanism of work stress on Chinese local undergraduate teachers’ turnover intention (1–9, 14, 15, 17–20).

### 2.1 Work stress and turnover intention

This study proposes that work stress is an important factor that induces university teachers’ turnover intention. The work stress of local undergraduate university teachers includes teaching stress, research stress, economic stress, daily trivial affairs, etc. (21, 22). This study argues that there are two key reasons why work stress triggers turnover intention. First, high work stress often reflects high job demands, and fulfilling higher job demands tends to accelerate the loss of employees’ resources. Conservation of Resources Theory (COR) suggests that in each iteration of the resource loss spiral, resource reserves are sharply reduced and university teachers first adopt a defensive mode to reduce or avoid investing resources, which in turn generates a willingness to leave (23). Second, university teachers also have to expend more limited emotional and cognitive resources to cope with work stress. In order to preserve existing resources or reduce further loss of resources, university teachers are more likely to adopt a defensive attitude of reducing resource investment, engage in more job procrastination behaviors, and have a stronger turnover intention. Therefore, the following hypothesis is proposed:

*H1:* Work stress positively predicts turnover intention, that is, faculties with higher work stress are more likely to have a stronger turnover intention.

### 2.2 The mediating effect of job burnout

Why do university teachers have the intention to resign in the face of work stress? This study proposes that job burnout mediates the relationship between work stress and turnover intention. Burnout is seen by Durham as an extreme form of work stress, a product of irreconcilable stress responses, and by Maslach as a stress response that includes emotional exhaustion, dehumanization, and a lack of personal effectiveness (24). However, it is worth pointing out that burnout is not equivalent to work stress, as the two have essential differences. Work stress is studied from a one-dimensional perspective, while job burnout is studied from a multidimensional perspective. It not only includes emotional reactions to work stress, but also includes evaluations of others and self-evaluations caused by work stress. Stress response is usually generated by the perceived inconsistency between the individual’s perceived job demands and his or her ability, while job burnout is generated by the perceived inconsistency between the individual’s perceived commitment to the job and the rewards obtained from the job, with emotional factors taking a low priority. Work stress itself does not necessarily lead to burnout, but if individuals are under prolonged work stress that cannot be resolved, and there are no buffer resources and no support systems during this period, then these irreconcilable stresses can develop into burnout (25). Given that burnout is a comprehensive physical and psychological response in stressful situations, the definition of teachers’ burnout is determined in the context of Maslach’s three-dimensional theoretical model: teachers’ burnout refers to an extreme reaction when teachers cannot successfully cope with work stress, and is a state of emotional, attitudinal, and behavioral exhaustion that teachers experience over a long period of stressful experiences (26). When faced with these negative experiences, university teachers try to alleviate their feelings of conflict and negative emotions through self-regulation behaviors, which will consume a large amount of valuable self-regulation resources. Moreover, this type of depleted resource is limited and usually not immediately reversible, which may easily lead to excessive depletion of self-regulation resources, ultimately forming a spiral of resource loss.

In addition, faced with work stress, university teachers need to make trade-offs in resource allocation between in-role and extra-role tasks, and such trade-offs can also deplete university teachers’ self-regulation resources. Emotional exhaustion, as a typical negative experience of job burnout, is an important influencing factor for employees to exit from organizations (27). When employees are physically and emotionally exhausted for a long period, their sense of belonging to the organization is diminished and they think of leaving the organization (28, 29). Emotionally exhausted employees lack enthusiasm for their work, have a sense of frustration, and are unable to devote time and energy to their work (30), which makes them more likely to become bored with their current job and the organization and leads to leaving. Withdrawal is a typical way to reduce the psychological cost of emotional exhaustion (31), and chronically exhausted employees tend to overestimate the importance of both avoidance and withdrawal coping strategies, and are highly likely to withdraw from their current work environment (32).

Emotionally depleted employees become less able to resist damage and may intentionally leave their current organization in order to avoid further physical and psychological damage (33). Research has shown that turnover intention is a negative result of emotional exhaustion continuing to develop to a certain extent (34). Conservation of Resources Theory (COR) emphasizes that individuals are more concerned about the extent of resource depletion than resource acquisition. Individuals in a state of burnout adopt a strength-preserving attitude and reduce their attention and effort in order to prevent the momentum and magnitude of the spiral of resource loss from continuing to grow, thus resulting in a willingness to resign. In addition, previous studies have shown that individuals in a state of job burnout have cognitive biases and believe that they lack control over the external environment and are prone to problems related to self-regulation failure, leading to avoidance, which is a form of self-regulation failure. Following this logic, this study predicts that local undergraduate university teachers in a state of burnout may be more likely to leave their jobs. Therefore, the following hypothesis is proposed:

*H2:* Job burnout mediates the relationship between work stress and turnover intention, that is, work stress may influence faculties’ turnover intention by increasing their job burnout.

### 2.3 The moderating effect of self-efficacy

Since work stress can cause burnout among university teachers, under what conditions can this adverse effect be mitigated? To answer this question, under the framework of Conservation of Resources Theory (COR), this study introduces an individual characteristic variable that is closely related to individual resources, namely teachers’ self-efficacy, to explore its possible moderating effect in the process of work stress affecting job burnout. According to Bandura, self-efficacy refers to an individual’s expectation of his or her ability to perform a behavior in a given situation, and it includes two components: outcome expectation and efficacy expectation. Outcome expectation refers to an individual’s speculation about what kind of outcome his or her behavior may lead to, while efficacy expectation refers to an individual’s subjective judgment about his or her ability to implement a certain behavior. According to Bandura’s theory, self-efficacy emerges as a cognitive mediator of an individual’s behavior, and an individual’s self-efficacy expectation reflects the nature and scope of his or her behavior, especially it reflects the level of effort and persistence that an individual puts forth when facing difficulties. R.S. Bhagat et al. argued that individuals with more positive self-beliefs are relatively less negatively affected when facing occupational stress (35). Jex found that self-efficacy plays a very important role in individuals’ coping with stressors (36). These findings corroborate Bandura’s view that individuals with high levels of self-efficacy usually do not perceive stressors as threats to themselves and are more likely to respond positively to stress, while individuals with low levels of self-efficacy experience self-doubt, lower achievement requirements, or even give up halfway once they encounter difficulties. In addition, the study by Kirsch et al. showed that individuals with high levels of self-efficacy take active measures to prevent stress before it occurs. Based on the views of many scholars on work pressure and self-efficacy, Xu Xiaodong believes that the mechanism of self-efficacy on work stress is mainly realized through the adoption of positive coping strategies, which may be mainly reflected in two aspects: willingness to control work and choice of coping strategies (37).

Self-efficacy, as an important resource of individuals, not only helps individuals to actively seek various resources, but also compensates for the over-consumed self-regulation resources, thus alleviating job burnout caused by work stress. Specifically, according to the investment principle of Conservation of Resources Theory (COR), individuals with high self-efficacy will make full use of positive psychological resources to obtain benefits, and further invest resources to avoid or recover from the loss of resources (38). Therefore, in the face of work stress, university teachers with a strong sense of self-efficacy are good at mobilizing psychological resources to construct an understanding of work stress from a positive perspective and alleviate negative experiences; in addition, they are also adept at acquiring work environment resources to balance the conflict between work stress and job tasks. With the easing of negative experiences and a sense of role conflict, university teachers’ self-regulation activities caused by work stress are reduced, and the consumption of self-regulation resources is reduced accordingly, which eventually alleviates the job burnout of university teachers. On the contrary, university teachers with low self-efficacy tend to protect existing resources and are not good at actively acquiring new resources from the university, thus it is harder to have sufficient psychological resources or work resources to cope with work stress, and they are prone to produce strong adverse reactions that cannot be relieved, which intensify the degree of job burnout. Therefore, the following hypothesis is proposed:

*H3:* Self-efficacy can buffer the positive effects of work stress on job burnout, that is, university teachers with high self-efficacy may experience relatively lower levels of burnout when faced with work stress.

### 2.4 Moderated mediation

Per the Conservation of Resources Theory (COR), work stress triggers teachers’ burnout in local undergraduate universities in China, which further stimulates teachers’ turnover intention. However, whether work stress triggers job burnout depends on teachers’ comparison of the value of acquiring the inputs and outputs of the resources in their work in colleges and universities. Self-efficacy can influence teachers’ value comparison process, and this paper hypothesizes that self-efficacy can influence the strength of the mediating effect of job burnout. In practice, it is often observed that there is a high level of teacher burnout among local undergraduate colleges in China. Theoretically, in the face of the same work stress, teachers with high self-efficacy are confident in investing resources to obtain good work performance, thus weakening the mediating effect of job burnout on turnover intention, and then teachers are less likely to leave. On the other hand, teachers with low self-efficacy are less confident in their ability to cope with work stress, which will strengthen the mediating effect of burnout on the willingness to leave, and they are more likely to escape from the local undergraduate colleges and universities. Therefore, the following hypothesis is proposed:

*H4:* Self-efficacy moderates the mediating effect of job burnout in the relationship between work stress and turnover intention.

**Figure 1:**
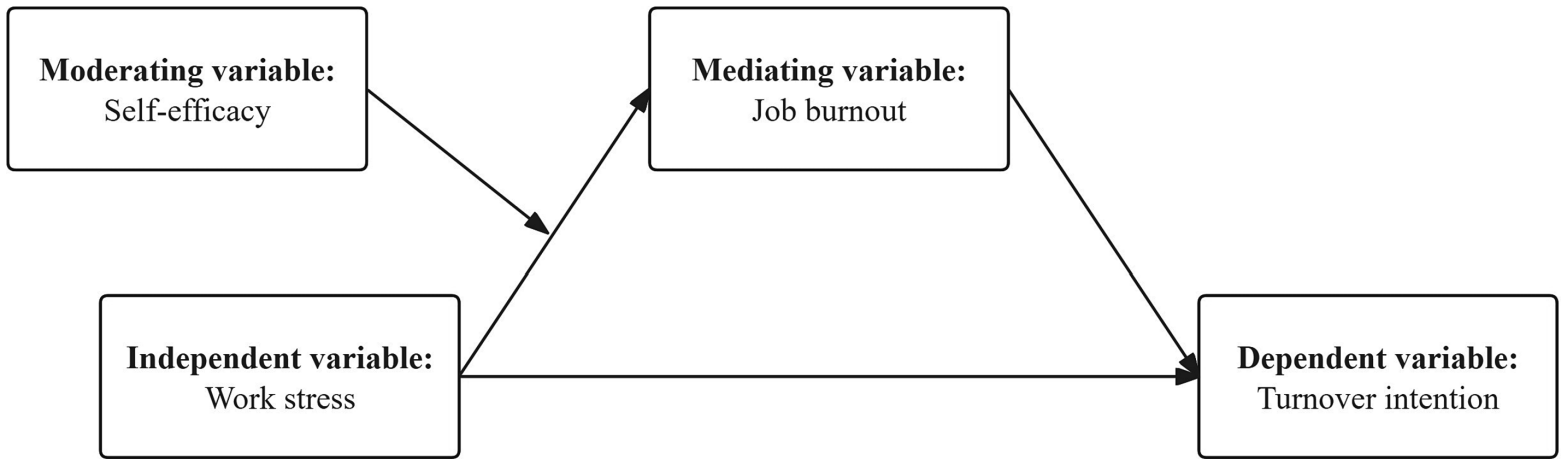
Concept of the moderated mediation model.

To sum up, this study aims to study the relationship between work stress, job burnout, and turnover intention of faculties in Chinese local universities, and examine the mediating role of job burnout and the moderating role of self-efficacy. The hypothesis model is shown in Figure 1.

## 3 Methods

### 3.1 Participants

This study distributed a faculty survey questionnaire to undergraduate institutions nationwide in May–June 2022. Faculties filled out the questionnaire by logging in to the online survey platform and answered all the questions before submitting them. The returned questionnaires were screened and the following four types of invalid questionnaires were excluded: first, questionnaires with less than 118 s of answer time were considered invalid; second, questionnaires with obvious regularity of answers were considered invalid; third, questionnaires with obvious logical problems, such as sleeping for more than 24 h and being older than 100 years, were considered invalid; fourth, questionnaire with wrong answers to the lie detection questions is regarded as invalid. After excluding the above four types of invalid questionnaires, 7,565 local undergraduate university teachers carefully finally filled out the questionnaire, with an effective response rate of 76.7%. Among them, 62.0% were female; 38.0% were male; 10.5% of them hold the professional title of full professor, 32.8% had the title of associate professor, 45.5% hold the intermediate professional title, and 11.2% hold the junior professional title; The proportion of university teachers with the highest degree of doctoral degree is 28.9%; master’s degree accounted for 59.4%; bachelor’s degree accounted for 11.7%; in terms of job types, teaching-oriented-type teachers accounted for 45.7%, research-oriented-type teachers accounted for 2.5%, teaching & research-oriented-type teachers accounted for 51.8%.

### 3.2 Measure

In this study, the Liker five-point scoring method (consent evaluation) was used for all scales.

#### 3.2.1 Work stress scale

The Work Stress Scale was based on a revision of the 26-item Sources of Faculty Stress Scale originally developed by Leung et al. (39). The scale has been published and widely used, ensuring its reliability and validity. Considering that the survey in this study was conducted in Chinese and that the questionnaire and scale have high scientific requirements for accuracy, the back-translation method was used in this study to translate this scale (40). The method followed a translation-back translation procedure to ensure the accuracy of translating from the original English scale into Chinese. Because this study measures the impact of work stress on work outcomes of local undergraduate university teachers, the specific scale questions were designed to accurately and realistically measure the work stress of local undergraduate university teachers by adding the statement “Please based on your actual feelings about various aspects of university work stress.” Examples include “overloaded with teaching tasks,” “stressful in writing and publishing papers/books,” “conflicting demands,” “teaching schedules are too tight” and “inadequate research grant,” etc. (Cronbach’s *α* = 0.95).

#### 3.2.2 Job burnout scale

The Job Burnout Scale is based on the MBI Burnout Inventory developed by Maslach et al. (41). This inventory is currently the gold standard for measuring occupational burnout. There are three forms of the MBI scales: the service industry version of the MBI-HSS (Human Services Survey), the education version of the MBI-ES (Educators Survey), and the general version of the MBI-GS (General Survey). The Burnout Scale for Local Undergraduate University Teachers is a 12-question scale selected from the MBI-ES (42), which inquiries about the true feelings of local undergraduate university teachers in their work. This scale adopts a five-point Likert rating, with 1 representing “never,” 2 representing “rarely,” 3 representing “occasionally,” 4 representing “often,” and 5 representing “always.” Item examples include “Work makes me feel exhausted,” “I treat certain students and colleagues as emotionless objects,” “Work makes me more and more numb,” and “Work makes me more and more indifferent to people” etc. (Cronbach’s α = 0.91).

#### 3.2.3 Turnover intention scale

The Turnover Intention Scale consists of one item that asks local undergraduate university teachers about their willingness to leave their jobs by Farhjl et al. (43). This scale adopts a five-point Likert scoring method, with 1 being “very small” and 5 being “very strong.” The specific test item is “My turnover intention is (very small 1 2 3 4 5 very strong).”

#### 3.2.4 Self-efficacy scale

The self-efficacy scale uses the 18-question scale of Te-Sheng Chang et al. (44). The sample questions such as “I believe I can teach according to the level of my students,” “I believe I can use information technology effectively to improve teaching,” “I have confidence in inspiring and maintaining students’ learning motivation,” “I believe I can utilize a variety of assessment methods to evaluate students’ learning results” and “I believe I can nurture a pleasant learning environment” etc. (Cronbach’s *α* = 0.91).

#### 3.2.5 Control variables

Gender, age, rank, teaching experience, education background, and type of position of university teachers were controlled.

### 3.3 Data analysis

This study first used confirmatory factor analysis to test the reliability and validity of the scale, followed by descriptive statistics and correlation analysis of the variables, and finally used a moderated mediation model of latent variables to explore the relationship among work stress, job burnout, self-efficacy, and turnover intention. In model analysis, we used the latent moderated structural equation (LMS) method to construct the moderated mediation model. Given that the LMS method cannot output conventional model fit indicators such as RMSEA, CFI, and SRMR, it is necessary to judge the model fit of the baseline model that does not involve the product term of the independent and moderating variables, and then to determine whether the fit of the LMS model is acceptable by comparing the AIC indicators of the LMS model and the baseline model (45). Subsequently, the bias-corrected Bootstrap method was further used to test the mediating effects, moderating effects, and moderated mediating effects in the model.

## 4 Results

### 4.1 Confirmatory factor analysis

In order to ensure the reliability and validity of the research tool, this study first used Mplus 8.3 to conduct a confirmatory factor analysis including 15 first-order factors. The results showed that the fit index of the measurement model was good: χ^2^[1070] = 17760.31, *p* < 0.001, RMSEA = 0.04, CFI = 0.96, NNFI = 0.95, SRMR = 0.04. The loading values of each factor item were greater than 0.60 (*p* < 0.001), indicating that the research tool had good construct validity; the AVE values of all factors were greater than 0.50, indicating that the convergent validity was good; the square root of AVE were all greater than the correlation coefficient between factors, indicating that the discriminant validity was good; the Cronbach’s α and combination reliability of all factors were greater than 0.70, indicating that the reliability of the factors was good. Overall, the reliability and validity of this research tool were ideal and suitable for further analysis.

### 4.2 Descriptive statistics and correlation analysis

Stata17.0 was used for descriptive statistics, normal distribution test and correlation analysis, and the specific results are shown in Table 1. In terms of work stress, university teachers scored higher than the theoretical median in only research stress (M = 3.52), while in role stress (M = 1.99), organizational stress (M = 2.40), teaching-research balance stress (M = 2.73), teaching stress (M = 2.66), student quality stress (M = 2.55), and competence stress (M = 2.28) were lower than the theoretical median, indicating that the overall work stress of university teachers was relatively low, and it was mainly research stress. In terms of job burnout, teachers’ emotional exhaustion (M = 2.31) and depersonalization (M = 1.62) were also lower than the theoretical median, indicating that teachers’ overall job burnout was relatively low and mainly manifested as emotional exhaustion. In terms of self-efficacy, teachers’ curriculum design efficacy (M = 4.37), teaching strategy efficacy (M = 4.24), classroom management efficacy (M = 4.36), interpersonal relationship efficacy (M = 4.40), learning assessment efficacy (M = 4.34), and teaching technology efficacy (M = 4.34) were higher than the theoretical median, indicating that university teachers have a high sense of self-efficacy in all aspects. In terms of turnover intention, teachers scored lower than the theoretical median, indicating that university teachers have a low turnover intention.

**Table 1:** Correlation matrix, reliability and validity, and descriptive statistics for each dimension of work stress, job burnout, self-efficacy, and turnover intention (*n* = 7,565).

		1	2	3	4	5	6	7	8	9	10	11	12	13	14	15	16	Cronbach’s α	CR	AVE	Loadings range
1.	RoS	0.82																0.86	0.86	0.67	0.72–0.88
2.	OS	0.75^***^	0.80															0.87	0.88	0.64	0.67–0.88
3.	ReS	0.43^***^	0.55^***^	0.84														0.91	0.91	0.71	0.81–0.89
4.	BS	0.48^***^	0.60^***^	0.66^***^	0.78													0.85	0.86	0.61	0.66–0.88
5.	TS	0.49^***^	0.61^***^	0.52^***^	0.61^***^	0.83												0.86	0.87	0.68	0.80–0.86
6.	SS	0.46^***^	0.57^***^	0.47^***^	0.48^***^	0.52^***^	0.84											0.87	0.88	0.70	0.80–0.89
7.	CS	0.45^***^	0.54^***^	0.42^***^	0.46^***^	0.46^***^	0.60^***^	0.86										0.89	0.89	0.73	0.79–0.91
8.	EE	0.41^***^	0.48^***^	0.36^***^	0.41^***^	0.44^***^	0.37^***^	0.36^***^	0.84									0.90	0.90	0.70	0.76–0.93
9.	DE	0.42^***^	0.40^***^	0.19^***^	0.31^***^	0.31^***^	0.30^***^	0.36^***^	0.62^***^	0.82								0.89	0.89	0.67	0.71–0.91
10.	CDE	−0.23^***^	−0.23^***^	−0.07^***^	−0.14^***^	−0.15^***^	−0.22^***^	−0.34^***^	−0.29^***^	−0.44^***^	0.95							0.96	0.96	0.90	0.94–0.96
11.	TSE	−0.23^***^	−0.26^***^	−0.13^***^	−0.17^***^	−0.18^***^	−0.28^***^	−0.38^***^	−0.30^***^	−0.40^***^	0.85^***^	0.91						0.94	0.94	0.84	0.90–0.93
12.	CME	−0.26^***^	−0.27^***^	−0.10^***^	−0.17^***^	−0.19^***^	−0.26^***^	−0.36^***^	−0.31^***^	−0.46^***^	0.85^***^	0.87^***^	0.91					0.94	0.94	0.84	0.89–0.93
13.	IRE	−0.24^***^	−0.24^***^	−0.07^***^	−0.15^***^	−0.16^***^	−0.22^***^	−0.33^***^	−0.28^***^	−0.46^***^	0.82^***^	0.80^***^	0.90^***^	0.94				0.96	0.96	0.88	0.93–0.94
14.	LSE	−0.25^***^	−0.27^***^	−0.10^***^	−0.17^***^	−0.18^***^	−0.25^***^	−0.36^***^	−0.30^***^	−0.45^***^	0.83^***^	0.84^***^	0.87^***^	0.89 ^***^	0.94			0.96	0.96	0.89	0.94–0.95
15.	TTE	−0.25^***^	−0.26^***^	−0.10^***^	−0.17^***^	−0.17^***^	−0.24^***^	−0.39^***^	−0.28^***^	−0.43^***^	0.79^***^	0.78^***^	0.82^***^	0.84^***^	0.87 ^***^	0.93		0.95	0.95	0.86	0.88–0.96
16.	TT	0.36^***^	0.37^***^	0.17^***^	0.22^***^	0.25^***^	0.25^***^	0.24^***^	0.35^***^	0.35^***^	−0.19^***^	−0.18^***^	−0.20^***^	−0.18^***^	−0.19^***^	−0.18^***^	-	-	-	-	-
	Mean	1.99	2.40	3.52	2.73	2.66	2.55	2.28	2.31	1.62	4.37	4.24	4.36	4.40	4.34	4.34	1.69				
	SD	0.86	0.97	1.08	1.03	1.05	0.87	0.84	0.88	0.73	0.69	0.73	0.69	0.68	0.69	0.71	1.03				
	Skewness	0.78	0.45	−0.42	0.33	0.35	0.28	0.43	0.38	1.23	−0.83	−0.64	−0.84	−0.92	−0.74	−0.78	1.41				
	Kurtosis	3.28	2.58	2.47	2.44	2.50	2.92	3.00	2.96	4.34	3.11	2.76	3.16	3.25	2.88	2.92	4.18				

The mean scores for each scale ranged from 1 to 5. The triangular matrix is the Pearson correlation coefficient, and the diagonal line is the square root of the average extracted variance (AVE). RoS, Role stress; OS, Organizational stress; Res, Research stress; BS, Teaching-research balance stress; TS, Teaching stress; SS, Student quality stress; CS, Competence stress; EE, Emotional exhaustion; DE, Depersonalization; CDE, Curriculum design efficacy; TSE, Teaching strategy efficacy; CME, Classroom management efficacy; IRE, Interpersonal relationship efficacy; TTE, Teaching technology efficacy; TT, Turnover intention. ^*^p < 0.05, ^**^p < 0.01, ^***^p < 0.001 (two-tailed).

Additionally, the results of the normality tests indicate that, with the exception of depersonalization (skewness = 1.23, kurtosis = 4.34) and turnover intention (skewness = 1.41, kurtosis = 4.18), all other variables exhibit skewness within the range of −1 to 1 and kurtosis within the range of 2–4, suggesting a close approximation to a normal distribution. Given that both depersonalization and turnover Intention demonstrate a slight rightward skew and a more concentrated distribution, it is proposed to employ the bootstrap method to correct for non-normality in the data in subsequent statistical analyses. This approach will provide a more robust estimation result.

The results of correlation analysis indicated that, on the one hand, among the internal dimensions of each latent variable, first, there was a moderate to strong positive correlation between the seven factors of work stress (0.42 ≤ *r* ≤ 0.75). Second, there was a strong positive correlation between the two factors of job burnout (*r* = 0.62). Third, there was a strong positive correlation between the six factors of self-efficacy (0.78 ≤ *r* ≤ 0.90). On the other hand, among the variables of work stress, job burnout, turnover intention, and self-efficacy, first, there was a moderate positive correlation between the seven stress factors and the two burnout factors (0.30 ≤ *r* ≤ 0.48). Second, there was a weak to moderate positive correlation between the seven stress factors and turnover intention (0.17 ≤ *r* ≤ 0.37). Third, there was a moderate positive correlation between the two burnout factors and turnover intention (*r* = 0.35). Fourth, there was a very weak to moderate negative correlation between six self-efficacy factors and seven stress factors (−0.39 ≤ *r* ≤ −0.07). Fifth, there was a weak to moderate negative correlation between the six self-efficacy factors and two burnout factors (−0.46 ≤ *r* ≤ −0.28). Sixth, there was a weak negative correlation between the six self-efficacy factors and turnover intention (−0.20 ≤ *r* ≤ −0.18).

The above correlation analysis results collectively showed that there was a strong positive correlation among the internal dimensions of the three latent variables: work stress, job burnout, and self-efficacy. Moreover, there was a positive correlation between work stress, job burnout and turnover intention, while there was a negative correlation between self-efficacy and work stress, job burnout and turnover intention.

Before conducting the moderated mediation model analysis, the Harman single-factor method was used to test for common method bias. Since the first factor explained 35.5% of the unrotated exploratory factor analysis, which has not reached half of the total explanatory power, the common method bias in this study was not severe (46). Therefore, the data in this study are suitable for further model analysis.

### 4.3 Moderated mediation model

**Figure 2:**
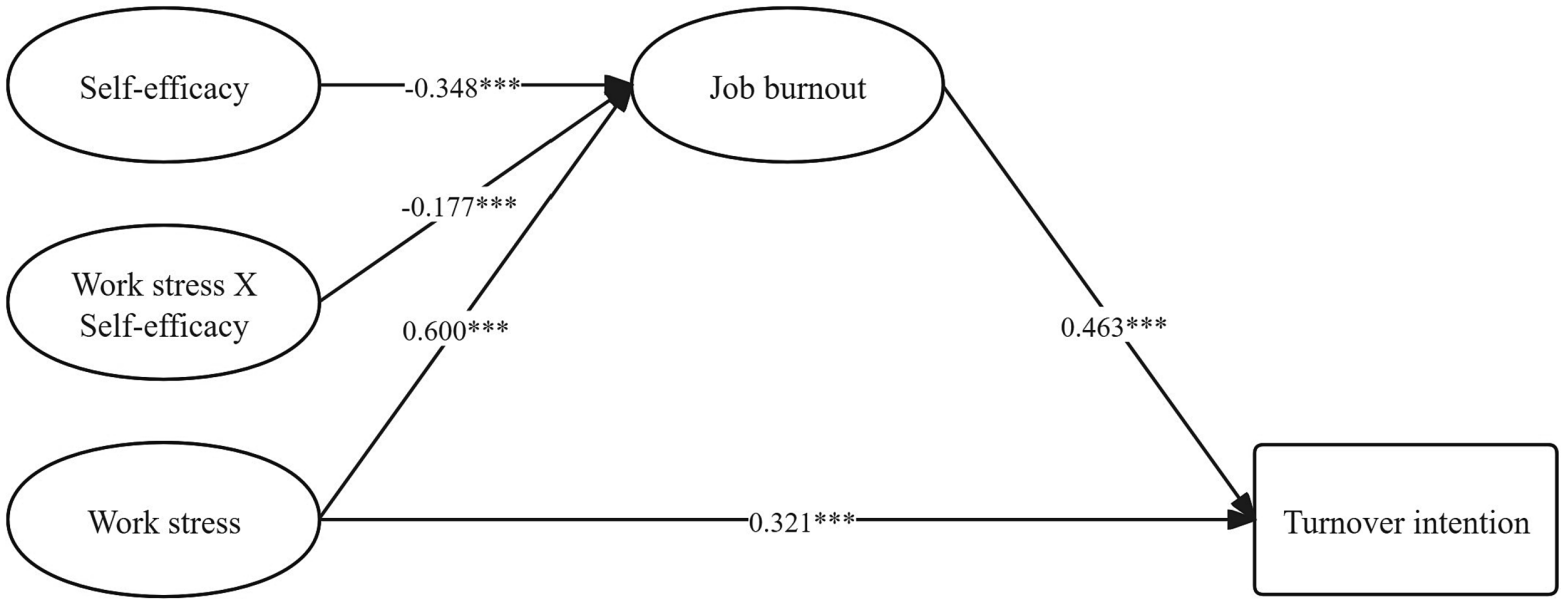
The moderated mediation model among work stress, job burnout, self-efficacy, and turnover intention. The image displays the non-standardized results. ^***^p < 0.001. For the sake of clarity and simplicity in the results diagram, the measurement indicators for each latent variable and control variables (gender, age, rank, teaching experience, education background, and type of position of university teachers) are omitted.

In this study, a benchmark model without the product item of work stress and self-efficacy was constructed first, and the results showed that the fitting index of the benchmark model was good: χ^2^[226] = 9564.03, *p* < 0.001, RMSEA = 0.07, CFI = 0.91, NNFI = 0.89, SRMR = 0.06, AIC = 207246.69. Subsequently, the LMS method was used to further construct a complete moderated mediation model, and the model results were shown in Figure 2. The AIC of this model was 207182.12, which was lower than the AIC value of the benchmark model, indicating that the moderated mediation model constructed in this study fit well and was acceptable. Each variable in the model explained 56.2% of the variation rate of job burnout and 23.5% of the variation rate of turnover intention. Overall, the hypothesis of this study was supported.

Firstly, among work stress, job burnout and turnover intention, work stress directly and positively predicted job burnout (B = 0.600, *p* < 0.001) and turnover intention (B = 0.321, *p* < 0.001), and job burnout directly predicted turnover intention (B = 0.463, *p* < 0.001). The results showed that work stress could positively predict turnover intention, and hypothesis 1 of this study was supported. In addition, when the moderating variable, self-efficacy, was at its mean value, the total effect of work stress on turnover intention was 0.599, comprising a direct effect of 0.321 and a mediating effect through job burnout of 0.278 (95%CI: 0.239–0.323). This mediating effect accounted for 46.4% of the total effect, indicating that work stress could indirectly predict turnover intention through job burnout, and hypothesis 2 of this study was supported (Table 2).

**Table 2:** The moderating effect of self-efficacy.

Path	Simple slope	95%CI	
		Lower	Upper
Work stress- > Job burnout	0.710 (Mean − 1 SD)	0.648	0.764
	0.600 (Mean)	0.556	0.644
	0.491 (Mean + 1 SD)	0.434	0.547
	0.220 (diff)	0.146	0.286
Work stress- > Job burnout- > Turnover intention	0.329 (Mean − 1 SD)	0.280	0.386
	0.278 (Mean)	0.239	0.323
	0.227 (Mean + 1 SD)	0.192	0.267
	0.102 (diff)	0.066	0.140

The table presents non-standardized results; All simple slopes across various levels of self-efficacy demonstrate statistical significance at the 0.001 level; diff refers to the difference between the simple slope of the group one standard deviation below the mean score (M − 1SD) and the simple slope of the group one standard deviation above the mean score (M + 1SD).

**Figure 3:**
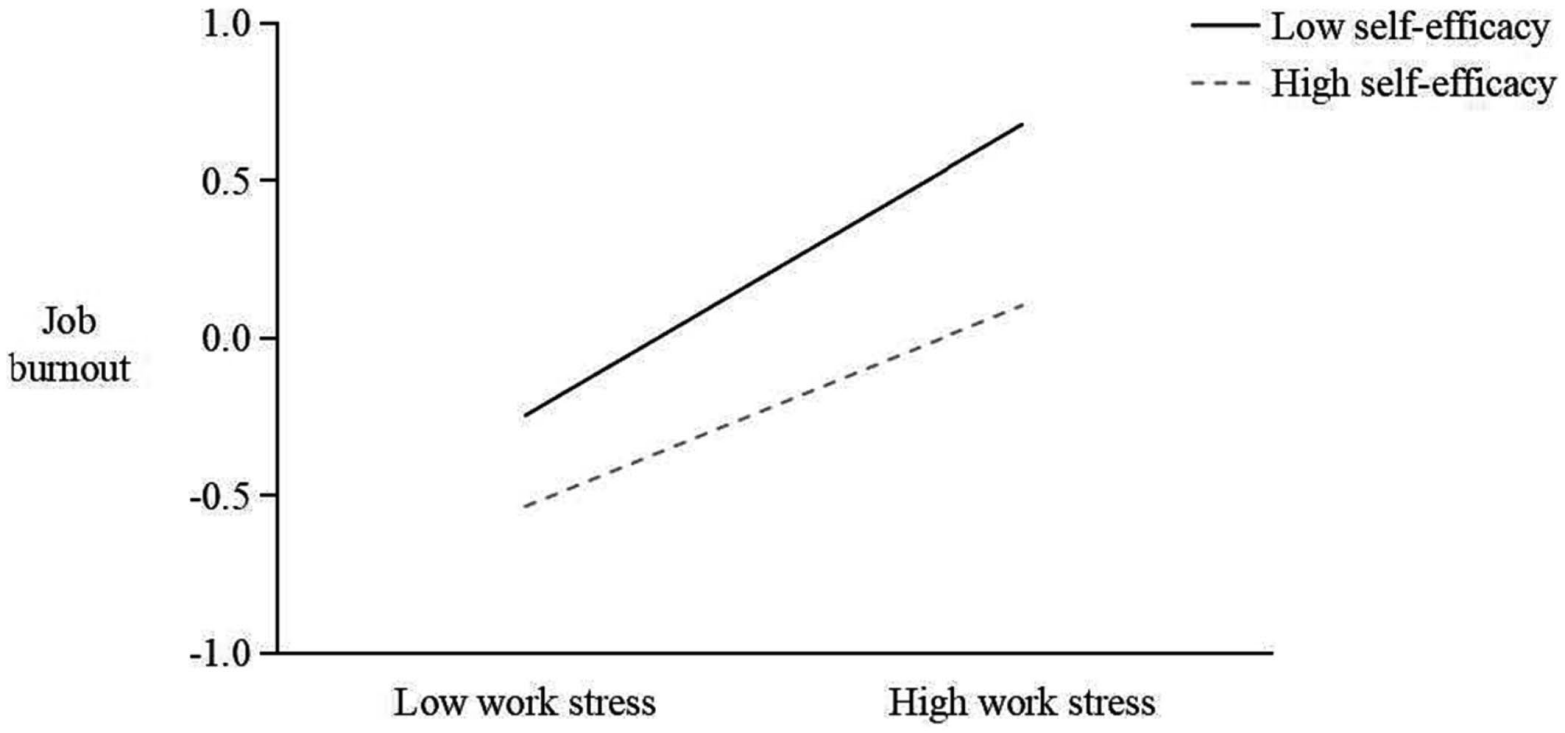
The moderating effect of self-efficacy in the relationship between work stress and job burnout.

Secondly, in terms of the adjustment of self-efficacy on the impact of work stress on job burnout, the interaction term of self-efficacy and work pressure negatively predicted job burnout (B = −0.177, *p* < 0.001), indicating that self-efficacy could buffer the positive impact of work stress on job burnout. A simple slope diagram was shown in Figure 3. A further simple slope test showed that when the self-efficacy was one standard deviation below the mean value (M − 1 SD), the predictive effect of work stress on job burnout (B = 0.710, 95% CI: 0.648–0.764, *p* < 0.001) was higher, and when the self-efficacy was one standard deviation higher than the mean value (M + 1 SD), the predictive effect of work stress on job burnout (B = 0.491, 95% CI: 0.434–0.547, *p* < 0.001) was lower. Moreover, the difference between the simple slopes of self-efficacy in the two situations was also significant (diff = 0.220, 95% CI: 0.146–0.286, *p* < 0.001). In conclusion, the above results showed that as the self-efficacy of teachers in local undergraduate universities increased, the predictive effect of work stress on job burnout decreased, and hypothesis H3 of this study was supported.

Finally, in terms of the moderated impact of self-efficacy on the mediating effect of job burnout, there is a significant differences in the mediating impact of job burnout across different levels of self-efficacy (diff = 0.102, 95%CI: 0.066–0.140, *p* < 0.001). When the self-efficacy was at low (M − 1 SD), the mediation effect of job burnout (B = 0.329, 95%CI: 0.280–0.386, *p* < 0.001) was higher, and when the self-efficacy was at high (M + 1 SD), the mediation effect of job burnout (B = 0.227, 95%CI: 0.192–0.267, *p* < 0.001) was lower. In conclusion, the above results show that as the self-efficacy of local undergraduate university teachers increased, the mediating effect of job burnout decreased, and H4 of this study was supported.

## 5 Discussion

Based on the Conservation of Resources Theory (COR), this paper constructs a moderated mediation model to explore the effects of work stress on faculty turnover intention in local undergraduate universities in China, and verifies the mediating effect of job burnout and the moderating effect of self-efficacy on the above direct and indirect effects. The findings of this paper enrich the discussion on the negative motivational effects of work stress, and promote the theoretical and practical fields to pay attention to the negative effects of work stress on teachers in local undergraduate universities in China.

### 5.1 The effect of work stress on turnover intention and the mediating role of job burnout

Studies have shown that teaching is a high-intensity and high-stress profession, and that high work stress can reduce teachers’ work efficiency, affect their physical and mental health, and hinder their professional development (47)^.^ Using turnover intention as an indicator, this study reveals that work stress has a direct positive predictive effect on turnover intention, expanding the scope of previous studies to a certain extent.

The relationship between work stress and job burnout is generally perceived in academia as a causal one. Vandenberghe and Huberman suggested that if an individual is under prolonged stress that cannot be resolved, and there are no buffer resources and support systems during this period, then the stress will gradually cause burnout (25).Li et al. (48) pointed out that work stress not only fails to effectively enhance the academic development of university teachers but instead leads to serious job burnout among them. Du argued that many university teachers are overwhelmed by teaching and research tasks, leading to significantly increased physical and emotional exhaustion (49). Ding et al. (50) identified high job demands and significant stress as the primary reasons for teacher burnout in universities. Joanne also highlighted that work stress is a key factor contributing to burnout, high turnover rates, and shortages among special education teachers in the United States (51). The present study found that the work stress of teachers in local undergraduate universities in China is significantly and positively correlated with job burnout, which is consistent with Kokkinos’ study (52).

The Conservation of Resources Theory (COR) considers resources as all things that are valuable to an individual, and the motivation for an individual’s behavior is to conserve and acquire valuable resources. When an individual perceives that resource depletion is unstoppable, or when invested resources do not yield the expected resource outputs, it will lead to stress, which will trigger the stress response (53). According to the Conservation of Resources Theory (COR), the likelihood that an individual will have access to high-value output resources will have a direct impact on the generation of stress. When an individual faces performance stress, there are two possible responses: to stop consuming resources in order to preserve them, or to continue to invest in existing resources in order to obtain other more valuable resources (54). How an individual acts depends on how he or she compares the value of resource inputs and outputs; when the value of the output resource is higher than the value of the input, the individual tends to put in a lower-value resource in exchange for a higher-value resource; when the value of the resource in return is lower than the value of the input, the individual tends to discontinue the depletion in order to conserve the resource. The loss spiral perspective in the Conservation of Resources Theory (COR) states that the initial loss of a resource triggers a further loss of the resource, and that the spiral of resource loss develops more rapidly and the negative effects are more intense. Work stress is the main cause of burnout. The Job Demands-Resources Model suggests that in an environment with high job demands and low job resources, individual employees are prone to burnout, which in turn leads to poor work outcomes (55). This study proves that job burnout mediates the relationship between work stress and turnover intention, revealing the cognitive path of “work stress—job burnout—turnover intention,” which supports these theories to a certain extent, makes up for the deficiencies of previous studies, and deepens the understanding of the intrinsic mechanism by which work stress affects the turnover intention.

The most prominent cognitive precursor to teacher turnover is their turnover intention (56), which refers to an individual’s psychological inclination to depart from the organization (57), directly triggering actual resignation behaviors (58). Research on factors influencing teacher turnover intention has predominantly focused on individual factors, compensation, job characteristics, and organizational management. Individual influencing factors encompass gender, teaching experience, marital status, among which novice teachers tend to exhibit a higher turnover intention (59). In terms of job characteristics, factors such as role stress, work–family conflict, professional identity, and job burnout also impact the level of turnover intention. Within organizational management, the support system proves pivotal in encouraging teachers to continue their educational roles, with higher perceived organizational fairness and identification reducing the likelihood of choosing to leave their positions. Furthermore, the decision to leave is often an outcome of the interaction between individuals and their surrounding environment. According to the Job Demands-Resources model, social support, serving as a job resource, effectively buffers the physiological and psychological effects of job demands, thereby reducing teacher turnover intention (60). Social support, defined as external assistance and protection (61), is crucial for university teachers and typically emanates from leaders, colleagues, family, and friends (62). Hence, social support is recognized as a significant protective factor in alleviating the inclination of university teachers to resign. External care and assistance enable teachers to approach their work more positively and optimistically, mitigating job burnout, fostering a higher degree of work engagement, and consequently lowering the turnover intention.

### 5.2 The moderating role of self-efficacy

This study found that self-efficacy of teachers in local undergraduate universities in China moderates the relationship between work stress and job burnout as well as the relationship between work stress and turnover intention. Overall, when the self-efficacy of teachers of local undergraduate universities in China is high, the role of work stress is weakened both in terms of job burnout and turnover intention, while when the self-efficacy of teachers of local undergraduate universities in China is low, the role of work stress is greatly enhanced both in terms of job burnout and turnover intention. This finding is also consistent with the results of related studies at home and abroad (63).

When the occupational stress caused by job demands is associated with a lack of coping resources, i.e., the job demands exceed the individual’s coping resources, then it will lead to the phenomenon of threat and damage, and the long-term imbalance will further develop occupational burnout. These coping resources can be external, such as social support, or internal, such as perceived self-efficacy, self-concept, role conflict/ambiguity, and other cognitive factors. This is also illustrated by the research findings that among the many coping resources, two are the most important: self-efficacy is the most influential internal resource and social support is the most significant external resource (64).

Ten Brummelhuis and Bakker pointed out that critical resources are the constraints under which individual employee stress processes occur and play an important moderating role. They further pointed out that self-efficacy, as an individual characteristic resource, is a key resource (65). Self-efficacy refers to an individual’s confidence or belief in his or her own abilities (66). Employees with high self-efficacy believe in their own abilities and are confident in their ability to accomplish their goals and tasks, and they will be more active in facing their goals and tasks and achieve high performance levels through high commitment (67). According to the Conservation of Resources Theory (COR), individuals’ behavior in response to stress depends on their value judgments of resources, and individuals are willing to invest low-value resources in exchange for high-value resources, thus realizing the transformation of resources in different fields and the growth of resource stock. Employees with high self-efficacy have more confidence and resources to cope with work pressure, and believe that they can obtain high performance rewards through their own efforts. Compared to employees with low self-efficacy, they place a higher value on their personal abilities and efforts, and are less likely to suffer from job burnout or to run away from their jobs. Therefore, for local undergraduate university teachers with high self-efficacy, the effect of work stress on turnover intention is weakened. Teachers with low self-efficacy do not have enough confidence in relying on their own efforts to achieve good performance, and feel constrained in terms of time, energy, and resources, so they are more likely to experience a spiral of resource loss, which in turn leads to job burnout. Therefore, for teachers with low self-efficacy, the effect of work stress on the turnover intention is enhanced. The moderating effect of self-efficacy found in this study also supports the above view, and is consistent with the findings of overseas studies that efficacy moderates the relationship between work stress and job burnout (68).

### 5.3 Theoretical implications

Firstly, this study expands on the research on antecedent variables of teachers’ turnover intention in higher education. Over the past few decades, China has experienced intensified organizational reforms, leading to a significant increase in personnel mobility between organizations, with unpleasant work experiences becoming a key driver of faculty turnover. During this period, Chinese higher education universities underwent various changes in organizational structure and management systems. These changes included the establishment of dual-level administrative structures with certain autonomy granted to colleges and departments, reforms in curriculum design such as the introduction of new courses and interdisciplinary programs, and an emphasis on practical teaching and encouraging student participation in research projects. While these reforms aimed to enhance the quality of higher education and align it with social and economic development, they also brought additional work pressures, particularly for university faculty, making work-related stress a significant factor influencing their work experiences. This study employs the COR theory as a theoretical framework to investigate the impact of work stress on turnover intentions among university faculty. The results of the empirical study indicate that work stress affects job burnout and turnover intention. This study expands the research on the antecedent variables of university teachers’ turnover intention and provides a new direction for the study of turnover behavior (69–72).

Secondly, based on the Conservation of Resources Theory (COR), this study introduces the intermediary variable of job burnout, trying to reveal the “black box” of the mechanism of the relationship between work stress and turnover intention. The findings show that job burnout plays a mediating role between work stress and turnover intention. This is because, in the face of work stress, local undergraduate university teachers often go through a self-regulation process, which will excessively consume self-regulation resources that can be used for follow-up work. In the state of resource depletion, university teachers often choose to preserve existing resources, and generate turnover intention.

Third, based on the theoretical framework of resource conservation, this study further reveals the individual contingency mechanism by which work stress affects turnover intention: the negative moderating effect of self-efficacy. This study believes that local undergraduate university teachers with high self-efficacy can withstand higher work pressure, often actively seek ways to supplement the consumed self-regulation resources, and restrain or avoid the generation of turnover intention by improving self-control ability. The above results not only support the constructive effect of self-efficacy in helping local undergraduate university teachers cope with work stress, but also further deepen the academic circle’s understanding of the boundary conditions of work stress affecting university teachers’ turnover intention.

### 5.4 Management implications

Firstly, teachers and administrators of local undergraduate universities in China should arrange the work of each post reasonably, pay attention to the adverse consequences of work stress, and reduce the generation of turnover intention. It is worth noting that sometimes there may be certain reasons for assigning non-compliance tasks to university teachers. Then the administrators should explain the reasons or express apologies to the relevant faculties in order to reduce their work stress and weaken their negative impact (73). Secondly, local undergraduate universities should prevent the loss of teachers’ self-regulation resources. This requires the organization to build reasonable and effective communication channels between superiors and subordinates, and to fully understand the working status of university teachers through online and offline communications. Finally, since self-efficacy can alleviate the depletion effect of work stress, and the research results show that the self-efficacy level of female teachers is higher than male teachers, it would be beneficial to implement measures that focus on cultivating the self-efficacy of male teachers. This could help reduce the turnover intention among university faculty. In terms of professional titles, the self-efficacy of the professor group and associate professor group is higher than that of the junior professional title group, which reminds university administrators to pay special attention to junior professional title faculties, and to improve and cultivate the self-efficacy of young teachers such as teaching assistants who have just entered the university. In terms of job types, the self-efficacy of teaching-oriented-type teachers is lower than that of teaching and research-oriented-type teachers and research-oriented-type teachers. Local undergraduate universities in China can guide teachers to develop into teaching and research-oriented-type and give full play to the advantages of a virtuous cycle of teaching and scientific research that complement each other.

### 5.5 Limitations

The shortcomings of this paper are as follows: firstly, the study lacks multi-source data. Future research can collect data from different time points and different sources to reduce the impact of common method bias. Secondly, future research can consider whether there are other mediating and regulating mechanisms between work stress and turnover intention. According to the theory of cognitive evaluation of stress, stress can be divided into obstructive stress and challenging stress. Future research can further refine the different mediating mechanisms of different stresses; it may be interesting to explore some states or situational moderating factors, such as paying attention to the moderating role of leadership factors (e.g., narcissistic leadership). Third, in terms of control variables, considering that facing work stress may cause local university teachers to experience negative emotional problems, and the process of repairing these negative emotions may also trigger ego problems with mediation failures that generate turnover intention. Therefore, it is necessary to use the emotional state of local university teachers as a control variable in future research to exclude the impact of negative emotions on the results of this study. Fourth, there are other theories and variables that can be used to elucidate the moderating role of self-efficacy. For example, Eccles’ Expectancy-Value Theory identifies expectancy for success and subjective task value as two important motivators for choice, performance, and persistence (74). Self-perception of competence explains people’s expectations of success, which refers to the belief that challenging tasks can be performed well (i.e., self-efficacy). Subjective task value refers to the importance people place on the tasks they plan to undertake. In future research, the variables of expectancy for success and subjective task value can be incorporated based on Expectancy-Value Theory to examine their effects on university teachers’ turnover intention.

## 6 Conclusion

The main findings of this paper are as follows.

1. The predictive effect of work stress on job burnout and turnover intention among local undergraduate teachers: this study reveals that work stress was effective in predicting burnout and turnover intention. Teachers experiencing higher levels of work stress are more prone to emotional exhaustion and professional identity disintegration, consequently leading to higher turnover intentions. Quality enhancement in higher education necessitates attention and intervention in addressing work stress among teachers in local undergraduate universities. This is crucial not only for the individual well-being of teachers but also for ensuring the stability of organizational structures in higher education institutions.
2. The complex relationship between work Stress, job burnout, and turnover intention: work stress among teachers in local undergraduate universities positively influences both job burnout and turnover intention, with job burnout partially mediating the relationship between work stress and turnover intention. Faced with heightened work stress, teachers are prone to experiencing physical and mental exhaustion, resulting in reduced work efficiency, ultimately leading to job burnout and reinforced turnover intentions. Therefore, to alleviate work stress among university teachers, it is imperative for administrators to follow the principles of job-person fit, rationally allocate work positions, and thereby effectively reduce turnover intentions.
3. The moderating role of self-efficacy in the relationship between work stress and turnover intention: the results indicate that self-efficacy significantly moderates the relationship between work stress and turnover intention among teachers in local undergraduate universities in China. When self-efficacy is high, the impact of work stress on turnover intention is relatively low. Conversely, teachers with lower self-efficacy are more likely to be affected by work stress and strengthen turnover intentions. This finding contributes beneficially to existing theories by emphasizing the importance of enhancing teachers’ self-efficacy to better cope with career changes, adapt to environmental shifts, overcome work stress, and consequently reduce turnover intentions.

## Data availability statement

The raw data supporting the conclusions of this article will be made available by the authors, without undue reservation.

## Ethics statement

The studies involving humans were approved by the Ethics Committee of Xiamen University (ECXMU-2022036). The studies were conducted in accordance with the local legislation and institutional requirements. The participants provided their written informed consent to participate in this study.

## Author contributions

SP: Conceptualization, Writing – original draft. SW: Formal analysis, Writing – original draft. RJ: Investigation, Writing – review & editing. JG: Conceptualization, Supervision, Writing – review & editing. JN: Writing – original draft, Data curation.
